# A multi-center, randomized, clinical trial comparing adhesive polyurethane foam dressing and adhesive hydrocolloid dressing in patients with grade II pressure ulcers in primary care and nursing homes

**DOI:** 10.1186/1471-2296-14-196

**Published:** 2013-12-21

**Authors:** Mireia Guillén-Solà, Aina Soler Mieras, Antònia M Tomàs-Vidal

**Affiliations:** 1 Primary Health Care-Mallorca: Research Unit. Health Care Services of Balearic Isles, IB-Salut, Palma de Mallorca, Balearic Islands, Spain; 2 Stem-center. USP-PalmaClinic, Palma de Mallorca, Balearic Islands, Spain; 3 Pressure Ulcer Advisory Group of the Balearic Islands, Palma de Mallorca, Balearic Islands, Spain

**Keywords:** Pressure ulcers, Pressure sore, Hydrocolloid dressing, Polyurethane foam dressings, Healing process

## Abstract

**Background:**

Pressure ulcers (PrUs) are ischemic wounds in the skin and underlying tissues caused by long-standing pressure force over an external bone or cartilaginous surface. PrUs are an important challenge for the overall health system because can prolong patient hospitalization and reduce quality of life. Moreover, 95% of PrUs are avoidable, suggesting they are caused by poor quality care assistance. PrUs are also costly, increasing national costs. For example, they represent about 5% of overall annual health expenses in Spain. Stages I and II PrUs have a combined prevalence of 65%. According main clinical guidelines, stage II PrUs (PrU-IIs) are usually treated by applying special dressings (polyurethane or hydrocolloid). However, little scientific evidence regarding their efficacy has been identified in scientific literature. Our aim is to assess the comparative efficacy of adhesive polyurethane foam and hydrocolloid dressings in the treatment of PrU-IIs in terms of healed ulcer after 8 weeks of follow-up.

**Methods/design:**

This paper describes the development and evaluation protocol of a randomized clinical trial of two parallel treatment arms. A total of 820 patients with at least 1 PrU-II will be recruited from primary health care and home care centers. All patients will receive standardized healing procedures and preventive measures (e.g. positional changes and pressure-relieving support surfaces), following standardized procedures. The main outcome will be the percentage of wounds healed after 8 weeks. Secondary outcomes will include cost-effectiveness, as evaluated by cost per healed ulcer and cost per treated patient and safety evaluated by adverse events.

**Discussion:**

This trial will address the hypothesis that hydrocolloid dressings will heal at least 10% more stage II PrUs and be more cost-effective than polyurethane foam dressings after 8 weeks.

**Trial registration:**

This trial has been registered with controlled-trials number ISCRCTN57842461 and EudraCT 2012-003945-14.

## Background

Pressure ulcers (PrUs) are ischemic wounds in the skin and underlying tissues, caused by continuous pressure from friction or shearing between an external surface and a bone or cartilaginous surface. Long-standing pressure can reduce capillary blood flow and lead to cell death, necrosis and broken tissue, or to additional serious complications, including osteomyelitis, sepsis, contractures, atrophy and psychological disorders [1,2]. These complications may delay mobilization and active rehabilitation, as well as reducing the ability of patients to live active and independent lives. PrUs can affect patients in all health care settings, provoke pain and discomfort, decrease quality of life and even increase morbidity and residence time in healthcare institutions [3,4].

PrUs can also increase direct and indirect healthcare costs and provide a negative image of healthcare institutions, attributable to deficits in the quality of care, especially since 95% of PrUs are preventable [1,2]. A national study has estimated that expenditures related to the onset of PrU amounts to approximately 5% of the annual healthcare spending in Spain [3,4].

A national prevalence study in 2006 by the GNEAUPP (Spanish acronym of National Group for the Study and Advice in Pressure Ulcers and Chronic Wounds) showed a wide variability in PrUs prevalence among types of institutions. PrUs have been reported to occur in 3.73% of patients in domiciliary care, 8.24% of hospitalized patients and 6.1% of patients in nursing homes. Moreover, 2.1% of patients with PrUs were aged 0 to 45 years, 6.4% were aged 46 to 64 years and 87.4% were aged ≥ 65 years [5]. Within each category, PrU incidence was very variable, ranging from 3-29% in hospitalized patients. Moreover, PrUs have been observed in 66% of elderly individuals (risk population) with femur fractures [4-7].

The European Pressure Ulcer Advisory Panel (EPUAP) has classified PrUs in four stages. Stages I and II are more frequent, with a combined rate of about 65% [5], although it differs among studies [8-15]. The EPUAP has defined stage II PrU (PrU-II) as involving a partial loss in skin thickness, affecting the epidermis and/or dermis. These are considered superficial ulcers, clinically manifesting as abrasions or blisters [8,16].

Prevention is the best treatment for PrU. However, despite Good Clinical Practice Guidelines (GCP), [17-20] which report the effectiveness of preventive activities (adequate nutrition, effective pressure relief, positional changes, management of incontinence and elimination of shearing and friction forces), there is little scientific evidence supporting the efficacy of these measures in avoiding further problems related to PrUs [1-3].

In contrast, evidence has suggested that PrUs healing in a moist environment, using special or modern dressings, is more cost effective than traditional cure or dry-to-wet dressings (i.e. dressings that do not maintain a moist environment) because the former stimulate cell proliferation. In addition, special dressings act as a barrier against bacteria, absorb excess wound fluid, reduce pain during the healing process and create the right conditions (moist environment) for healing or scarring [1,3,18-20].

The benefits of a moist environment have led to the development of a flowering variety of synthetic dressings (special or also called modern dressings). Use of these dressings depends on the availability of resources, PrU stage and morphology, and the presence of infection and/or necrosis [1,3,18-20].

Although GCP guidelines indicate that the special dressings most frequently used to treat PrU-II are hydrocolloid and polyurethane foam dressings. To date, however, systematic reviews and clinical trials have established a poorly and scarcely evidence of their effectiveness. Although studies have shown benefits of polyurethane dressings, their benefits did not differ significantly from those of hydrocolloid dressings. In addition, these studies involved patients with various wound types (pressure ulcers and venous ulcers) and different PrU stages, which may have affected the results [1,3,18-21].

To overcome these limitations, the present study will compare two types of dressings in the treatment of PrU-IIs, both which are recommended and are part of habitual clinical practice [1,3,19,20]. However, due to the wide variability in both types of dressing, this study will compare the adhesive versions of polyurethane and hydrocolloid dressings.

### Aim and hypothesis

The main hypothesis of this study is that hydrocolloid dressings would heal at least 10% more stage II PrUs than polyurethane foam dressings over 8 weeks of follow-up in patients with PrU-II.

The aim of the proposed protocol is to compare the efficacy of adhesive polyurethane foam dressings and adhesive hydrocolloid dressings in the treatment of PrU-II. The primary objective will be to assess the percentage of wounds healed after 8 weeks.

Secondary objectives include assessments of:

improvements in the clinical efficacy of both dressings, as measured by ulcer area (cm [2]), exudates, type of tissue (PUSH Scale) and time to healing (epithelial tissue).

treatment efficacy, as measured by PrU-II resolutionwithin study time.

safety, as determined by adverse events (AE) attributed to both dressings.

direct costs of both dressings, as measured by cost per healed ulcer and cost per treated patient.

convenience of dressing use, as determined by adherence properties, management of the dressing, comfort, pain during the healing procedure, number of dressings used and perilesional skin state.

## Methods and design

### Study design

This will be a multicenter, randomized trial evaluating and comparing the efficacy of the adhesive polyurethane foam and hydrocolloid dressings in patients with PrU-II within the context of habitual prevention measures.

### Setting and eligibility criteria

This study will recruit patients who receive primary health assistance, including patients able to attend the primary health center or receive health assistance in homes, nursing-homes and non-acute long term hospitalization centers. Each patient must present with at least one PrU-II.

#### Inclusion criteria

Age ≥ 18 years.

Confirmed diagnosis of PrU-II. If a patient has more than one PrU-II, only the largest diameter ulcer will be assessed. Other PrU-II will receive the best treatment elected by the study nurse.

#### Exclusion criteria

Stage I, III or IV PrU only.

Non classifiable PrU.

Surgical treatment prior to PrU and/or PrU in previously irradiated areas.

Participation in another clinical trial within 3 months of study entry.

Allergy or hypersensitivity to materials in the study dressings.

Signs of PrU basal infection (sepsis/bacterial), cellulitis or osteomyelitis. Patients successfully treated for infection can be included if the PrU can be classified as stage II.

Venous ulcers and/or diabetic feet.

Type I diabetes.

Situations of extreme severity and/or agony; e.g. patients in terminal phase with <3 points on the Braden scale and/or a life expectancy < 1 month.

### Recruitment

Each primary health center or nursing home will have one or two study nurses to assess eligibility criteria, to invite potential candidates to participate in the clinical trial and to carry out all protocol procedures (see section on Interventions).

Potentially eligible patients will be recorded, as well as reasons for exclusion. All included patients will be given a patient information sheet and an informed consent form. Signing of the latter is essential for study inclusion.

If a patient’s clinical condition makes signing of the informed consent form impossible, a relative or guardian will be responsible for signing; however, those patients must clearly indicate orally their willingness to participate in the study.

Any included patient who drops out or is lost during the clinical trial process will be recorded on the appropriate form.

### Randomization

Participants will be enrolled consecutively by their study nurse. Each participating center will have a unique randomization list. This sequential randomization will be generated in blocks of 12, up to a maximum of 24 recruited patients per center (see Additional file 1: Figure S1).

### Sample size

Assuming that 30% of patients in the polyurethane arm will show healing of PrU-II at 8 weeks, an alpha risk of 5%, a beta risk of 20% with bilateral contrast and a loss to follow-up of 15%, 410 individuals will be required in each treatment arm to detect a ≥10% difference in healed ulcers [22,23].

### Blinding

Due to an inability to mask the appearance of the two dressings, it will only be possible to mask the final evaluation of PrU.

### Interventions

Participants will be randomized to hydrocolloid or polyurethane dressing. All patients will receive a standardized preventive intervention to reduce pressure and undergo a PrU healing cure process.

***Transversal interventions*** are designed for pressure relief and positional changes, favoring an optimal evolution of stage II PrU (PrU-II). Adequate relief of pressure forces (shearing or friction) requires changes in position of bedridden or sitting patients and the use of pressure-relieving support surfaces (PRSS).

Due to their structure, PRSS can reduce pressure forces, as well as heat and humidity, increasing patient comfort. PRSS can cover the entire body or only part of it and can be found in different sizes and types: mattresses, mats or cushions. However, it is important to highlight that the use of PRSS does not mean ignoring other healing procedures, including changes of position, skin care and good nutritional state. Rather, PRSS use only serves as a complement to the healing process [1].

To standardize changes in position and pressure reduction, study nurses will follow a national GCP recommendations [1]. Recommendations for frequency of position changes, as well as specific recommendations for bedridden and sitting patients, must be taken into account. Each study nurse or caregiver will be trained to properly implement the recommendations in the aforementioned GCP.

***Healing cure process*** addresses the cleansing process and prevention of infection. Standardization of this process can be accomplished using recommendations from the reference GCP. Briefly:

*The wound must be cleaned every time the study dressing is changed*. The first step is to thoroughly irrigate the wound bed with saline solution to remove detritus, bacteria and remains of any previous treatment. A 20 ml syringe of saline solution adapted with a 0.9 × 25 needle or a 19 mm catheter. Local antiseptics (e.g. povidone, chlorhexidine) and skin cleaners should be avoided because they are cytotoxic to new granulating tissue and their habitual use can have systemic effects due to absorption.

*The drying procedure must be also delicate*. Rough material (such as gauzes or sponges) can induce small traumas on the wound bed, increasing the risk of infection and interfering with the healing process. Wound edges should be dry and clean and wound beds moist. Care should be taken to avoid damaging healthy tissue during cleansing and drying procedures.

*Bacterial infection should be prevented*. An aseptic technique should be used if possible, including the use of clean gloves. Proper healing and debridement procedures can minimize the risk of infection. If a patient presents with more than one PrU-II, the most contaminated one should be left to the end (i.e. perennial area).

It is important to isolate and remove waste and contaminated materials in accordance with established precautions to avoid cross contaminations.

If there is leakage of exudates, lack of adherence or any situation suggesting loss of study dressing, additional (secondary) dressings will be used. If this additional support is inadequate, the study dressing will be changed following the same procedure and be registered in the CRF.

### Study dressings

#### Polyurethane foams

##### Description and features

These dressings derive from polyurethanes and have a hydrophilic structure. They present with a high capacity of autolytic debridement and absorption of exudates; and keep the wound bed from drying out, without leaving residuals or decomposing. In addition, they avoid leakages, stains and odors, keep periwound skin intact, reduce frictional forces and do not produce traumas when removed.

##### Indications

These dressings are recommended for all stages of PrU (with moderate or high exudates). Frequent monitoring and changes may be necessary when PrUs become infected.

##### Contraindications

These dressing cannot be combined with antiseptics (e.g. iodine, chlorhexidine, hypochlorite, ether or hydrogen peroxide), oxygenated water or sodium hypochlorite because all of the latter can destroy the dressing [1,3].

#### Hydrocolloids

##### Description and features

These dressings are made of carboxymethylcellulose (CMC) and other hydrocolloids (elastomers), adherent substances or hydroactive compounds, providing absorption capacity. They are covered with a polyurethane layer, giving them occlusive or semi occlusive properties. In addition, they absorb exudates and necrotic residuals by forming a gel with special color and odor characteristics, creating a slightly acid environment with bacteriostatic properties. Moreover, they reduce friction forces.

##### Indications

These dressings are recommended in non-infected PrU stages I, II and III; slough ulcers and those with necrotic tissue (as an autolytic debriding agent) and granulation phase or epithelialization healing process.

##### Contraindications

These dressings should not be applied to infected ulcers or those in which bones or tendons are observed. Ether and aggressive antiseptics should be avoided when using these dressings [1,3].

### Variables

#### Main outcome variables

The main efficacy outcome of the study is rate of PrU-II healing or ulcer epithelialization tissue (scored on the PUSH scales as 0). Two digital photographs will be taken, one at the beginning and the other at the end of treatment, and sent to the expert panel committee from Balearic Islands (GAUPP) for evaluation. This committee will be blinded to treatments of individual patients.

### Secondary outcome variables

#### Efficacy

*Changes in the basal PUSH scale,*[24-27] ulcer area (in cm [2]) at the end of treatment, exudates and tissue type. This information will be collected weekly until the end of the study or until the PrU-II heals.

*Changes in ulcer area in cm*[2] will be measured using ImageJ software [28,29]. Two photographs, taken at the beginning and end of treatment (8 weeks or when the wound heals), will be evaluated by members of the expert panel committee. A <10% difference in ulcer area measured by two evaluators will be considered ‘agreement’ and the final measure will be the average of these measurements. A ≥10% difference in ulcer area between the two evaluators will be considered ‘disagreement’, requiring measurement by a third investigator, with the final measure being the average of the two closest measurements. This method has been used in other studies to evaluate changes in ulcer area [30].

Cost-effectiveness (partial cost-effectiveness evaluation): cost per proportion of healed ulcers and cost per treated patient. In both treatment arms, the healing process registry must reflect information regarding dressings and material used, including cost per unit dressing, cost per secondary dressing, additional material used for each patient (e.g. saline, gauzes, globes, tubing, syringes), and time spent by each nurse performing these procedures (mm:ss).

Dressing convenience (patient comfort) [31-33] will be evaluated by both patients and study nurses using Likert scales ranging from 1 (worst outcome) to 5 (best outcome) to assess the following measures:

*Patients:* adherence to dressing; pain at dressing removal; pain during application of dressing; overall comfort; and time during the healing process

*Study nurses:* adherence to dressing; ease of applying the dressing; ease of removing the dressing; absorption by the dressing; perilesional skin condition, as assessed by erythema; perilesional skin condition, as assessed by maceration; and time during the healing process.

### Withdrawal criteria

Patients will be removed from the trial if any of the following conditions are met:

•Infection and/or progression of PrU-II

•Disease progression, requiring discontinuation of the investigational product treatment regimen or study requirements.

•An adverse event (AE) that requires discontinuation of the investigational product or study procedures.

•Voluntary withdrawal

•Loss of follow-up

Protocol deviations will be registered. In case of a serious protocol deviation, the research team will assess the possibility of withdrawing the patient from the study.

Study discontinuations must be registered in the CRF, and, where possible, tests and procedures usually performed at the final visit (visit 8 or end of study) should be carried out. Patients who withdraw due to AEs must be followed until the end of the study (8 weeks), and if necessary until AE resolution.

### Work plan

Before the trial begins, the study protocol will be presented to all research team members in a special meeting. A training session lasting 3–4 h will include a review of the inclusion and exclusion criteria, provide instructions regarding interventions and the procedure to use to fill out the Case Report Form (CRD), as well as assessing the ethical requirements for the trial. The protocol to be used to collect information from each patient at each study visit will include (see Additional file 2: Table S1):

•**Selection visit (screening)**

Assessment of inclusion/exclusion criteria

Signing informed consent form

•**Visit 1 (baseline).***If patients agree to participate in the study at the selection visit, all scheduled activities for visit 1 can be performed at the selection visit. If patients require time to think about participating, these scheduled activities will be performed at visit 1.*

Randomization process

Collection of demographic data, including date of birth, sex, and place of usual care

Risk factors for PrU, using the Braden scale

PUSH Scale registry, including area of each lesion (width × length), volume of exudates and tissue type

PrU photographic registry

Healing process registry: reason to change dressing, time treated by the nurse, materials used

Training about transverse care (positional changing and pressure reduction)

Recording of concomitant medications

Recording of adverse events

•**From Visit 2 to Visit 7 (Follow-up)**

Assessment of inclusion/exclusion criteria

Assessment of transverse interventions, from each patient’s diary and nursing files

PUSH Scale registry, including area of each lesion (width × length), volume of exudates and tissue type

Healing process registry: reason to change dressing, time treated by the nurse, materials used

Recording of concomitant medications

Recording of adverse events

•**Between visits.***The study protocol includes 8 study visits. If patients require a change of dressing, between visits,it must be recorded in the CRF, with the following information collected.*

Date of dressing change

Reason for dressing change (exudates, lack of adherence, other reasons)

Time treated by the nurse (mm:ss)

Materials used

•**Visit 8 or End of study**

PUSH Scale registry: diameter of each lesion (width × length), volume of exudates and tissue type.

Completion of the dressing scale comfort instrument by patients and study nurses

Braden scale

PrU photographic registry

Recording of concomitant medications

Recording of adverse events

This study will last 8 weeks (8 visits) or until the ulcer heals, whichever comes first. The protocol for Visit 8 can include 3 different situations:

1. *PrU-II heals before week 8 (visit 8).* If complete healing is observed at an earlier visit, the nurse will perform the activities described for that Visit plus those described for Visit 8.

2. *PrU-II does not heal by week 8 (visit 8)*. The nurse will perform all the activities described for Visit 8.

3. *The patient presents some withdrawal criteria before week 8 (Visit 8).* The nurse will perform all the activities described for the earlier visit, as well as those described for Visit 8.

### Statistical analysis

#### Intention to treat analysis (ITT)

All statistical calculations will be based on ITT analysis, including all patients randomized, whether treated or not, and including all patients who have withdrawn prematurely. This approach reduces any bias that may occur when participants not receiving assigned treatments are excluded from analysis. All tests will be two-sided and α-values of 0.05 will be considered statistically significant.

#### Descriptive analysis of the patients

The trial involves a descriptive analysis of the baseline characteristics of patients in both treatment arms. Quantitative variables will be reported in measures of central tendency (mean or median) and the corresponding standard deviation or interquartile range. Qualitative variables will be reported as proportions.

#### Baseline comparisons

Baseline qualitative variables in the two treatment arms will be compared using chi square tests (χ^2^), and quantitative variables using Student’s t-tests. Non-parametric tests will be used when distributions are not normal.

#### Comparative analysis

In a bivariate analysis, a χ^2^ test will be used to assess whether the percentage of healed PrU-IIs differs significantly in the two treatment arms. Mean changes in PrU size will be compared using Student’s t-tests for normal distributions or the Mann-Whitney U test against the usual null hypothesis (non-normal distributions): (initial area-final area)*100/initial area). In addition, 95% confidence intervals will be calculated to assess the clinical significance of treatment.

A logistic regression model adjusted for potential confounders will be performed when patients are not equally distributed in both treatment arms. Relative and absolute risk reduction and number needed to treat, defined as the estimated number of ulcers needed to be treated with the chosen dressing for one additional ulcer to be healed will be estimated, along with their corresponding 95% CIs.

Time to healing in the two treatment arms will be calculated by the Kaplan-Meier method and compared using the log-rank test. A Cox regression model of proportional risks will be used when the models require adjustment for any statistically significant baseline variable.

Finally, a partial economic evaluation will be performed, including direct costs such as number of dressings used per patient, additional dressings used, materials used, labor cost per cured ulcer and further treatment cost. The overall differences in mean costs and effects between treatments will be calculated using Student’s t-tests.

### Ethics

The trial has been approved by the Balearic Island Ethic Committee. It will be performed by qualified nurses. The rights and welfare of patients will be respected throughout the trial. All involved patients will be informed, verbally and in writing, of the trial objectives, risks and possible benefits. Signed informed consent form will be required from each patient. The trial will respect and follow the standards of good clinical practice enshrined in the Declaration of Helsinki [34,35].

## Discussion

PrUs are an important health problem, not only due to their high incidence and prevalence but to their effects on health and quality of life. Of these lesions, about 40% have been categorized as stage II [36,37].

PrU-II treatment consists mainly of the application of polyurethane and hydrocolloid dressings, which are recommended and part of habitual clinical practice. However, little is known about the relative efficacy of these two types of dressings.

Among available data, highlights a recent systematic review evaluating the effectiveness of modern dressings in the treatment of venous ulcers [21] found that hydrocolloid dressings were the most common type, evaluated in 27 of 45 (60%) clinical trials (CTs). Most of these CTs compared hydrocolloid dressing with other types of dressings or compared different types of hydrocolloid dressings.

The review included a meta-analysis of four CTs, involving 311 patients, [38-41] comparing the effectiveness of hydrocolloid and polyurethane foam dressings for venous ulcers, with the primary outcome being the total number of healed ulcers in 8–12 weeks. However, that analysis found no statistically significant difference in the healing rates with dressings (pooled relative risk (RR) 0.98; 95% CI, 0079 to 1.22).

Similarly, another meta-analysis of three CTs [23,42,43] comparing the effectiveness of hydrocolloid dressings with preparations of polyurethane foams in treating 129 patients with PrUs [20], found no significant between group difference (RR, 0.82; 95% CI, 0.57 to 1.17).

Most previous studies comparing polyurethane and hydrocolloid dressings have methodological shortcomings, reducing the value of their results and conclusions. These included small sample sizes, resulting in low statistical power, as well as a lack of patient follow up, absence of ITT analysis, different outcome measures, and the inclusion of various wound types and different PrU stages.

Furthermore, among the many types of PrU treatments and dressings currently in use are silicone hydrogels, hydrocellular polyurethanes and polyurethane foams, hydropolymeric silicone and foams, hydrocolloids, and antimicrobial bioactive collagen (collagenase). However the use of most is based on little scientific evidence [3].However, scientific evidence is required to optimize the treatment of PrU-II, and pressure ulcers in general.

This study has been designed to avoid the methodological problems encountered in other studies and to provide better evidence for optimal PrU-II treatment. Our protocol includes training sessions for nursing staff involved in the conduct of the trial, and standardizing data collection.

Identification of optimal dressings can improve patient quality of life and decrease treatment costs. It won’t be able to succeed unless we don’t stop and try to shed light on this health problem with the resources which are at our disposal and are recommended in the main GCP guidelines with little evidence about them.

## Abbreviations

PrU: Pressure ulcers; PrU-II: Stage II pressure ulcers; EPUAP: European pressure advisory panel; GCP: Good clinical practice guidelines; CT: Clinical trial; AE: Adverse events; ITT: Intention to treat analysis

## Competing interests

The authors declare that they have no competing interests.

## Authors’ contribution

AS and MG conceived the project. However, the main idea for the research project came from the Pressure Ulcer Advisory Group of the Balearic Islands (*Grupo Asesor de Úlceras por Presión de las Islas Baleares:* GAUPP), whose main representative is AMT. In addition, some GAUPP members constitute the expert panel responsible for confirming the occurrence of PrU-II and its healing (or not) at the end of the study. MH, AM, JM, CP and MAR are responsible for contacting the main study nurses, at primary health centers, nursing homes, and non-acute long term hospitalization centers AL is responsible statistical analyses and sample size calculations. MA and SM are responsible for the logistics of obtaining and delivering study dressings to primary health centers, nursing homes, and non-acute long term hospitalization centers. ME and JL have provided unconditional support and scientific knowledge to improve the study. Finally, MG was responsible for drafting this manuscript. All authors revised and approved the final version of the manuscript.

## Pre-publication history

The pre-publication history for this paper can be accessed here:

http://www.biomedcentral.com/1471-2296/14/196/prepub

## Supplementary Material

Additional file 1: Figure S1 Illustrations and Figures. Click here for file

Additional file 2: Table S1 Schedule of Events. Click here for file

## GAUPP-Expert Panel (Pressure Ulcer Advisory Group of the Balearic Islands)

Reyes Marín, GAUPP-Expert panel; Amélia Pérez, GAUPP-Expert panel; Conchita Rodríguez, GAUPP-Expert panel; Juana Fernández, GAUPP-Expert panel; Maria Dolores García, GAUPP-Expert panel; Antonia Fullana, GAUPP-Expert panel.

## Collaborators

Marisol Hernández, GAUPP-Expert panel; Maria Adrover Rigo (madrover@gmail.com), GAUPP-Expert panel; Jerònia Miralles (jmiralles@ibsalut.caib.es); Carmen Pata (cpata@ibsalut.caib.es), Maria Alemany (maria.alemany@ibsalut.caib.es); Sonia Martínez (sonia.martinez@ibsalut.es); Magdalena Esteva (mesteva@ibsalut.caib.es); Joan Llobera (jllobera@ibsalut.caib.es); Alfonso Leiva (aleiva@ibsalut.caib.es); Angélica Miguélez (amiguelez@ibsalut.caib.es).
