# Supplementary figures and images for: A multi-center, randomized, clinical trial comparing adhesive polyurethane foam dressing and adhesive hydrocolloid dressing in patients with grade II pressure ulcers in primary care and nursing homes
Source: BMC Fam Pract. 2013 Dec 21;14:196. doi: 10.1186/1471-2296-14-196 (PMC3907779; doi:10.1186/1471-2296-14-196)

## Annex I. ILLUSTRATIONS AND FIGURES

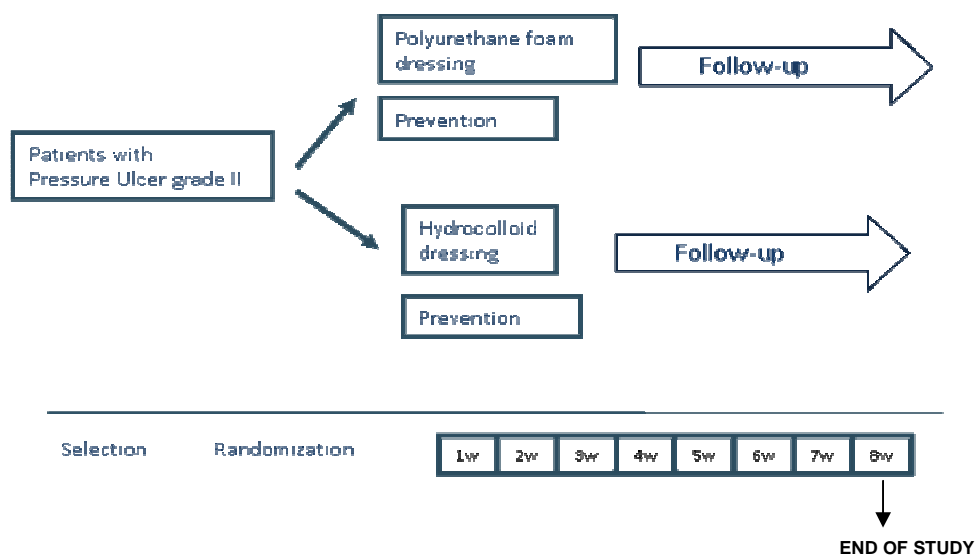

Supplement: Additional file 1: Figure S1 — Illustrations and Figures. [file 1471-2296-14-196-S1.pdf]
