# Supplementary material for: A multi-center, randomized, clinical trial comparing adhesive polyurethane foam dressing and adhesive hydrocolloid dressing in patients with grade II pressure ulcers in primary care and nursing homes
Source: BMC Fam Pract. 2013 Dec 21;14:196. doi: 10.1186/1471-2296-14-196 (PMC3907779; doi:10.1186/1471-2296-14-196)
Supplement: Additional file 2: Table S1 — Schedule of Events. [file 1471-2296-14-196-S2.pdf]

Annex II. Schedule of Events

	AT THE SAME TIME *								
	W. 0	W.1	W.2	W.3	W.4	W.5	W.6	W.7	W.8
	Screening V. (-1)*	V.1 (basal)	V.2	V.3	V.4	V.5	V.6	V.7	V.8 or End of study
Inclusion-Exclusion Criteria	x	x	X	x	x	x	x	x	x
Patient Information Sheet Informed Consent	x	x							
Demographic Data Registry		x							
Braden scale		x							x
PrU-II photographic registry		x							x
PrU-II basal characteristics		x							
PUSH scale registry		x	x	x	x	x	x	x	x
Randomization		x							
Transverse care**									
Positional changing			x	x	x	x	x	x	x
Reduction of friction			x	x	x	x	x	x	x
Prevention of infection			x	x	x	x	x	x	x
Reason for changing dressing									
Per protocol			x	x	x	x	x	x	x
Other reasons***			x	x	x	x	x	x	x
Time recorded (mm:ss)			x	x	x	x	x	x	x
Number of study dressings used and material needed			x	x	x	x	x	x	x
Comfort (patients/nurses)									x
Concomitant medications		x	x	x	x	x	x	x	x
Adverse Events		x	x	x	x	x	x	x	x

W: week; V: visit; CRF: Case Report Form; mm:ss: minutes and seconds
*Patients who agree to participate at the screening visit can undergo all of the procedures of Visit 1 at the same time. V1 could occur no later than 24 h after the screening visit.
**Positional changes, reduction of friction and prevention of infection should be recorded in each patient's diary and on the CRF.
*** Changes in dressing between study visits should be recorded on the CRF.
